# Machine Learning and Bayesian Network Analyses Identifies Psychiatric Disorders and Symptom Associations with Insomnia in a national sample of 31,285 Treatment-Seeking College Students

**DOI:** 10.21203/rs.3.rs-3944417/v1

**Published:** 2024-02-21

**Authors:** Adam Calderon, Seung Yeon Baik, Matthew H. S. Ng, Ellen E. Fitzsimmons-Craft, Daniel Eisenberg, Denise E. Wilfley, C. Barr Taylor, Michelle G. Newman

**Affiliations:** The Pennsylvania State University; The Pennsylvania State University; Nanyang Technological University, Rehabilitation Research Institute of Singapore; Washington University School of Medicine; University of California-Los Angeles; Washington University School of Medicine; Stanford University School of Medicine; The Pennsylvania State University

**Keywords:** Insomnia, psychiatric disorders, machine learning, Bayesian network analysis

## Abstract

**Background:**

A better understanding of the structure of relations among insomnia and anxiety, mood, eating, and alcohol-use disorders is needed, given its prevalence among young adults. Supervised machine learning provides the ability to evaluate the discriminative accuracy of psychiatric disorders associated with insomnia. Combined with Bayesian network analysis, the directionality between symptoms and their associations may be illuminated.

**Methods:**

The current exploratory analyses utilized a national sample of college students across 26 U.S. colleges and universities collected during population-level screening before entering a randomized controlled trial. Firstly, an elastic net regularization model was trained to predict, via repeated 10-fold cross-validation, which psychiatric disorders were associated with insomnia severity. Seven disorders were included: major depressive disorder, generalized anxiety disorder, social anxiety disorder, panic disorder, post-traumatic stress disorder, anorexia nervosa, and alcohol use disorder. Secondly, using a Bayesian network approach, completed partially directed acyclic graphs (CPDAG) built on training and holdout samples were computed via a Bayesian hill-climbing algorithm to determine symptom-level interactions of disorders most associated with insomnia [based on SHAP (SHapley Additive exPlanations) values)] and were evaluated for stability across networks.

**Results:**

Of 31,285 participants, 20,597 were women (65.8%); mean (standard deviation) age was 22.96 (4.52) years. The elastic net model demonstrated clinical significance in predicting insomnia severity in the training sample [R^2^ = .449 (.016); RMSE = 5.00 [.081]), with comparable performance in accounting for variance explained in the holdout sample [R^2^ = .33; RMSE = 5.47). SHAP indicated the presence of any psychiatric disorder was associated with higher insomnia severity, with major depressive disorder demonstrated to be the most associated disorder. CPDAGs showed excellent fit in the holdout sample and suggested that depressed mood, fatigue, and self-esteem were the most important depression symptoms that presupposed insomnia.

**Conclusion:**

These findings offer insights into associations between psychiatric disorders and insomnia among college students and encourage future investigation into the potential direction of causality between insomnia and major depressive disorder.

**Trial registration:**

Trial may be found on the National Institute of Health RePORTER website: Project Number: R01MH115128-05.

## Introduction

Sleep disturbance is often conceptualized as a transdiagnostic mechanism observed across a range of psychiatric disorders [1] and, in some cases, is even included as a diagnostic criterion [2]. Unlike earlier conceptualizations of insomnia as merely a symptom or consequence of other mental health issues, an emerging perspective suggests that sleep and other psychiatric disorders are intricately intertwined and bidirectional [3]. Such implications are not unexpected given that patients who report sleep-wake disorders, notably and most commonly, insomnia, exhibit higher rates of comorbidity, e.g., 40% of those with insomnia report having additional disorders as compared to 16.4% of those with no sleep difficulties [4]. Meta-analyses and reviews have found associations between sleep disturbance and most psychiatric disorders, including all anxiety disorders, depression, alcohol use disorder, and eating disorders [5–7]. Given the interrelationships between insomnia and several psychiatric disorders, further teasing apart its relations may help us understand important associations and their directionality.

Embracing such complexity requires a nuanced approach and the ability to aggregate disparate small variable effects to inform clinical outcomes. Unlike traditional statistical models (e.g., linear regression), machine learning engenders the opportunity to capture the simultaneous effect of all relevant predictors, even accounting for complex, interactive, or non-linear effects [8]. Particularly, supervised machine learning, such as elastic net regularization, possesses the capacity to predict outcomes of interest whereas minimizing the adverse effects of noisy data and reducing the probability of spurious, false positive associations [9]. Accordingly, elastic net regularization has been shown to lead to parsimonious models with greater stability and accuracy and with higher out-of-sample predictive performance (i.e., increasing the generalizability of the model to new patients) relative to linear regression [10]. Consequently, such algorithms have been exemplified in recent studies; for example, utilizing baseline data from a randomized controlled trial, Bard et al. [11] individually predicted functional impairment and the relative importance of depressive and anxiety symptoms among insomnia patients. Or, for instance, Lyall et al. [12] who employed actigraphy and mental health data from the UK Biobank to determine the most important sleep features (e.g., sleep duration, chronotype) related to depression and whether patients with poorer outcomes could be identified. Although the advantages of employing elastic net regularization are evident, disentangling directionality remains a challenge. Although there are interpretability frameworks such as the seminal SHAP (Shapley Additive exPlanations), which elucidates variable importance along with their directions [13], revealing the structure of relations and related emergent properties persists as a formidable task.

Network analysis is one methodological approach suited for such an endeavor, given its telos of disentangling the complex dynamics of self-reinforcing causal interactions between symptoms [14]. Broadly, in such an approach, a network comprises symptoms (nodes) and the associations between them (edges). In other words, an edge between nodes represents a conditional dependent relationship between two symptoms whereas keeping all other symptoms in the network constant [15]. Within this approach, hypotheses posit symptoms as causal agents that promote the development of other symptoms and, when unabated, go beyond a critical threshold and develop into a new harmful equilibrium known as a psychiatric disorder [16, 17].

Insomnia as a node or a set of nodes has appeared in many prior cross-sectional network analyses, providing snapshots of associations between symptoms. Extant studies include examining insomnia’s network structure itself [18–24] but also the relationships between single disorders, such as major depressive disorder (MDD) [25–28], post-traumatic stress disorder (PTSD) [29], psychosis [30], and schizophrenia [31], or between transdiagnostic factors, such as hyperarousal [32] or personality traits [33]. However, cross-sectional networks have also been developed between insomnia and multiple disorders, most commonly between MDD and generalized anxiety disorder (GAD) [11, 34–39] or with the further addition of PTSD [40], but also between prolonged grief disorder (PGD) and PTSD [41]. Most studies utilized the Graphical Gaussian Model (GGM), in other words, an undirected network of partial correlation coefficients, along with the graphical LASSO (Least Absolute Shrinkage and Selection Operator [42]), as a regularization technique to avoid spurious, false-positive edges. However, as pointed out by Williams and Rast [43] and further highlighted by McNally et al. [44], graphical LASSO was developed and optimized for high-dimensional settings with more variables than the number of participants, which often is not the case in typical network structures thus leading to unwarranted sparsity. Moreover, despite efforts, the conventional GGM approach employed in such studies make little inferences on directionality.

Conversely, Bayesian network analysis, such as directed acyclic graphs (DAGs), allows for estimating directed networks built on cross-sectional data. Although DAGs cannot confirm temporal precedence, such methods can provide preliminary clues to identify the direction of probabilistic dependence between edges [45]. In other words, if an edge originates from node X and connects to node Y (i.e., X → Y), node Y’s presence suggests or predicts node X’s presence more strongly than vice versa. Whereas the node considered the “parent” (X) might be present without its “offspring” (Y), the presence of the offspring indicates the presence of the parent. However, the assertion of causality is predicated on multiple conditions: these include, the absence of any bidirectional causal relations (such as X causing Y and Y causing X) or causal loops (such as X causing Y, Y causing Z, and Z causing X); and second, the absence of any significant variables missing from the dataset [44]. To our knowledge, two studies on insomnia and common comorbidities have taken such a Bayesian approach. In one of these studies, Zhang et al. [46] elucidated associations between insomnia and depression and health-related behaviors (e.g., internet use, physical inactivity, smoking, alcohol consumption) among adolescents in China. In the other study, Yu et al. [47] examined the relationships between sleep disturbance and mental health (e.g., anxiety, depression, loneliness, well-being, health attitudes) among adults in China. However, whether such associations can be generalized to other demographic groups or other psychiatric disorders requires further evaluation.

The current exploratory study thus aims to fill these gaps by examining the associations among insomnia and MDD, GAD, social anxiety disorder (SAD), panic disorder (PD), PTSD, anorexia nervosa (AN), and alcohol use disorder (AUD) among a nationally representative sample of treatment-seeking U.S. college students. Moreover, the present investigation extends a study by McCallum et al. [5], which used simple regression to examine the associations between sleep and nine mental health disorders. Often, the reliability and replicability of parameter estimates in cross-sectional network analyses are not considered and are, at the least, questionable [e.g., 48, 49–51]. In other words, echoing Epskamp et al. [15], the number of participants in network studies is typically insufficient to estimate the parameters included in the network accurately. Consequently, we used supervised machine learning to predict insomnia severity and network psychometrics to assess the directionality between comorbidities while also increasing power (cf. N = 3620). Furthermore, per recommendations by Bard and colleagues [11], we utilized causal search algorithms to elucidate the dynamics involved between insomnia and multiple psychiatric disorders. To do so, in line with Neal and Neal [52], who demonstrated the validity implications of in/exclusion of nodes when estimating networks and respective calls to action in needing conceptional justification of to-be modeled set of variables [52, 53]. The current investigation employed a straightforward, statistical approach to selecting edges in a graphical model. First, supervised machine learning determined the best subset of psychiatric disorders that led to optimal performance in predicting insomnia severity. Second, DAGs characterized the structure, relations, potential importance, and direction amongst the subset identified by supervised machine learning. Third, inspired by Bard et al. [11], who randomly partitioned their data into training and holdout samples to evaluate the replicability of their GGMs, we advanced such an approach to test the replicability of the resultant DAGs by computing structural distances between training and holdout samples to supplement traditional bootstrapped stability tests.

## Methods

### Participants

The current study is a secondary analysis of 39,194 treatment-seeking participants across 26 U.S. colleges and universities who participated in screening for an ongoing randomized controlled trial investigating the effectiveness of a transdiagnostic, coached mobile mental health intervention that uses population-level screening for engaging college students in tailored services for preventing and treating anxiety, depression, and eating disorders (clinicaltrial.gov; ID: NCT04162847). Participants were eligible for the screen if they were ≥ 18 years of age, enrolled at one of the 26 participating universities, provided informed consent to participate, and passed a one-item attention check. See Fitzsimmons-Craft et al. [54] for a more detailed description of the eligibility criteria. Participants were excluded for only previewing the survey (*n* = 1), not responding to (*n* = 5,513) or denying (*n* = 503) the consent for screening, being under 18 years old (*n* = 63), or not reporting their age (*n* = 1,154), not being an undergraduate student (*n* = 629) or their year in school had not been reported (*n* = 46). The final sample consisted of a national sample of 31,285 undergraduate students. All data for the present study were collected prior to selection for the randomized controlled trial or intervention delivery.

### Procedures

Students enrolled at participating universities received an email invitation to complete a brief survey on health and well-being between October 2019 and November 2021. Emails were sent to either the entire student population or a random subset of the student population and either to undergraduate students from all years (17 schools) or only years 1 or 2 (9 schools). Emails informed students that, based on their responses, they may be eligible for a subsequent study involving random assignment to conditions designed to support mental health. Emails included a link to an online screening survey via Qualtrics. Participating students were entered into a raffle to win one of several $100 gift cards. The study was approved by the institutional review board of all authors’ universities and administrators at each participating school.

### Measures

All models were based on data captured pre-intervention delivery and included insomnia, MDD, GAD, PTSD, SAD, PD, AN, and AUD.

Insomnia was assessed using the Insomnia Severity Index [ISI; 55]. The ISI has seven questions with 5-point Likert scale responses, which are summed to produce a total score between 0 and 28, with higher scores indicating greater insomnia severity. Cronbach alpha was .884. Its internal consistency, concurrent validity, and sensitivity to clinical improvements in insomnia patients are well established [56].

Major depressive disorder was assessed using the Patient Health Questionnaire-9 [PHQ; 57]. Participants reported frequency of depressive symptoms over the past two weeks on 9 items with four-point scales ranging from 0 (“Not at all”) to 3 (“Nearly every day”). The total score ranges from 0 to 27. Cronbach alpha was .877. Participants screened positive for probable MDD if they scored 10 or higher, maintaining a sensitivity of .88 and specificity of .85 [58].

PTSD was assessed using the Primary Care PTSD Screen [PC-PTSD; 59], which has total scores ranging from 0 to 4. Participants screened positive for probable PTSD if they scored three or higher, which demonstrated a sensitivity of .78 and specificity of .89 [59]. Cronbach alpha was .806.

GAD was assessed using the Generalized Anxiety Disorder Questionnaire-IV [GADQ; 60], maintaining a .82 specificity and .89 sensitivity, and has a total score ranging from 0 to 12. SAD was assessed using the Social Phobia Diagnostic Questionnaire [SPDQ; 61], maintaining a .85 specificity and .82 sensitivity, and has a total score ranging from 0 to 27 [61]. PD was assessed using the Panic Disorder Self-Report [PDSR; 62], maintaining a 1.00 specificity and .89 sensitivity, and has a total score ranging from 0 to 24 [62].Cronbach alpha was .856, .97, and .959, respectively. These measures all assessed full diagnostic criteria based on the Diagnostic and Statistical Manual of Mental Disorders, 5th edition (American Psychiatric Association, 2013). Participants screened positive for a disorder if they endorsed all diagnostic criteria. GADQ, SPDQ, and PDSR demonstrate strong test-retest reliability, good convergent and discriminant validity, and a kappa agreement of .67, .66, and .93, with structured interviews, respectively.

Anorexia nervosa (AN) was assessed by the Weight and Shape Concerns Scale [WCS; 63]. Total scores for the weight/shape concerns scale range from 0 to 100. Participants screened positive for probable AN if they scored 59 or higher and had a current body mass index ≤ 18.45, based on self-reported height and weight. Cronbach alpha was .797. These criteria have been used in prior online screening studies [64].

AUD was assessed using the Alcohol Use Disorders Identification Test Consumption [AUDIT; 65]. The instrument contains three questions about alcohol consumption with 4-point Likert scale responses, which are summed to obtain a total score ranging from 0 to 12. Cronbach’s alpha was .85. To identify probable AUD, we used the cutoff of 4 or higher for participants assigned male at birth and 3 or higher for participants assigned female or intersex. This system had .88 sensitivity and .75 specificity for males and .87 sensitivity and .85 specificity for females [66].

### Statistical Analysis

#### Pre-processing

The total data were randomly partitioned into a 70% split as a training set and a 30% holdout set to evaluate the final models in completely unseen new cases. Missing values for the included variables in our sample were low (< 7%). Nonetheless, to tackle missing data for all analyses, a machine learning approach for imputation was employed, specifically utilizing nonparametric missing value imputation via random forests facilitated by the R package *mice* [67]. Imputations were aggregated across 10 multiple imputed datasets, each with 100 iterations, to minimize biased error calculations and produce stable estimates. Random forest imputations were done separately for the training and holdout sets. Minimal recoding adjustments were made before each imputation to maintain the inherent relationships between variables (as recommended by van Ginkel et al. [68]). Moreover, to prevent “data leakage” of variable distributions between sets, all pre-processing steps were done separately for training and holdout sets. Topological overlap between node pairs was also screened for and removed if found via the “goldbricker” function within the R package *networktools* [69].

### Supervised machine learning (Elastic net regularization)

#### Elastic net development

Elastic net regularization is a form of conventional regression that combines both ridge and lasso norms to provide a penalization term to balance stability and parsimony. Accordingly, elastic net regularization was employed to constrain coefficients among collinear variables and minimize model overfitting, with the lambda hyperparameter determining the magnitude and the alpha hyperparameter regulating the balance between the two norms [9]. Tuning of alpha and lambda was conducted using resampling grid search and selected using repeated 10-fold cross-validation to minimize biased estimates of the true error and assess the stability of model performance [70]. 10-fold cross-validation partitions the sample into 10 subsets, 9 of which are used in the training process and then tested on the remaining subset [71]. This process is iterated for the remaining 10 subsets, building new models until each of the 10 subsets is used only once in the training and testing data. This procedure then repeats the 10 folds by 10 repeats for a total of 10 models. The final model is then averaged to produce a single estimate. Final alpha and lambda values were selected based on the smallest value of root mean square error (RMSE) and was used to estimate model coefficients.

In the current study, the elastic net model considered seven disorders (MDD, GAD, PTSD, SAD, PD, AUD, AN) as binary predictors (i.e., presence vs absence) and insomnia as a continuous outcome (i.e., total ISI score). Imbalance within the outcome was also addressed by applying the Synthetic Minority Over-Sampling Technique for Regression with Gaussian Noise [SMOGN; 72], which randomly undersamples high-frequency cases and oversamples rare cases using SmoteR and Gaussian Noise to generate a more balanced proportion of cases within the continuous outcome and improve prediction accuracy. Imbalance occurs when machine learning models favor predictions from high-frequency cases and ignore rare cases, given preferences for high accuracy, even if purely by chance. All analyses were conducted in R using version 4.3.1 using the caret package [73].

#### Elastic net evaluation

The cross-validated elastic net model built from the training sample was evaluated by being applied to individuals within the holdout sample to predict insomnia severity. Importantly, individuals within the holdout sample were not utilized as part of the development and tuning of the elastic net model. RMSE determined the accuracy of the model, i.e., the magnitude of the error. Lower values represent higher accuracy. The coefficient of determination (R^2^) was also used given evidence of R^2^ being the most informative metric within regression-based supervised machine learning [74]. R^2^ determined predictability, i.e., the proportion of variance within the outcome explained by the elastic net model. Values are interpreted as percentages and range from 0 to 1, with higher values representing higher predictability. The current study adopted the benchmark set by Uher et al. [75] who found an R^2^ of 6.3 or higher inferred clinical significance.

#### Elastic net feature importance

Methods for explainable artificial intelligence were run using SHAP (Shapley Additive exPlanation) values [13] to facilitate interpretability of the elastic net model. SHAP values assign a value to each feature that represents the average contribution of that feature across all possible combinations of features. The average SHAP value across all participants is 0, but the average absolute SHAP value informs about relative predictor importance.

### Bayesian networks (directed acrylic graphs)

#### Network estimation

DAG analyses were run via the hill-climbing algorithm from the R package, “bnlearn” [76] to determine the directionality and conditional dependencies among predictors. DAGs return a network comprising symptoms (nodes) and the relations between them (edges). To create the DAG, a bootstrap function computes the structural aspect of a network by adding edges, removing them, and reversing their direction to optimize a goodness-of-fit score (i.e., Bayesian Information Criterion [BIC]). This step determines whether an edge exists; however, it does not calculate the weights of the edges. To do so, we randomly restarted the process with different candidate edges linking different symptom pairs, perturbing the system. To ensure robustness, we used 50 restarts [as per Briganti et al., 45]) and 100 permutations [as implemented by McNally et al. 77, 78]. In the current study, we employed a Bayesian network via a completed partially directed acrylic graph (CPDAG), a type of Markov equivalence class that encodes identical conditional dependencies between DAGs and accounts for drawbacks of equivalent separate DAGs [79]. Insomnia was included in the DAG analyses as a single-sum score derived from the ISI representing insomnia severity, while item 3 from the PHQ [insomnia/hypersomnia] was removed to prevent multicollinearity.

#### Network stability

To verify the stability of the resultant network, we bootstrapped 10,000 samples, computed a network for each sample, and averaged all 10,000 networks to obtain the final resultant network. Following the reasoning of Briganti et al. [45], we first determined the structure of the network and then ascertained the direction of each edge. The bnlearn program computes a BIC value for each edge. The thickness of an edge corresponds to its absolute BIC value and, hence, its importance to model fit. The larger the absolute BIC value, the more damaging it would be to the model fit if one were to remove the edge from the network. Accordingly, high absolute BIC values indicate how important an edge is to the model that best characterizes the data structure. In line with Sachs et al. [80], if an edge ran from symptom X to symptom Y in at least 85% of the bootstrapped networks, this edge appeared in the final, averaged network. After which, if an edge ran from symptom X to symptom Y in at least 51% of the bootstrapped networks, its direction was depicted using an arrow pointing from node X to node Y. Accordingly, such significance thresholds promoted the stability of the final, averaged network and led to sparse networks that ensure genuine edges. Lastly, we then computed the identical network but had edge thickness reflect the probability that the depicted direction of the edge occurred.

#### Network confirmatory analysis

In summary, three steps were taken to ensure model stability: (1) random perturbations to avoid local maxima and optimize goodness-of-fit index (i.e., BIC values); (2) bootstrapping 10,000 different DAGs to determine strength and direction of the edges; (3) using significance thresholds outlined in Sachs et al. [80]. As a fourth step, a confirmatory analysis was run repeating steps one through three within the holdout sample and comparing structural distances to determine replicability. To compare the similarity between the training and holdout CPDAGs, Structural Hamming Distances (SHD) were used, which quantifies the number of changes between nodes, arcs, and the directions that must be made to a network for it to turn into the one that it is being compared [81]. In other words, calculating the true positive, false positive, and false negative arcs by comparing the training network to the holdout network, considered the “true” standard network. This allowed for testing whether the network estimation was roughly consistent across both data subsets, further suggesting replicability and confidence that results were not false positive or false negative.

## Results

### Sample characteristics

Screening sample characteristics for the entire sample are presented in Table 1. Most participants identified as female (63.4%), heterosexual (72.7%), white (65.7%), and non-Hispanic (67.5%).

### Elastic net regularization

**Figure 1:**
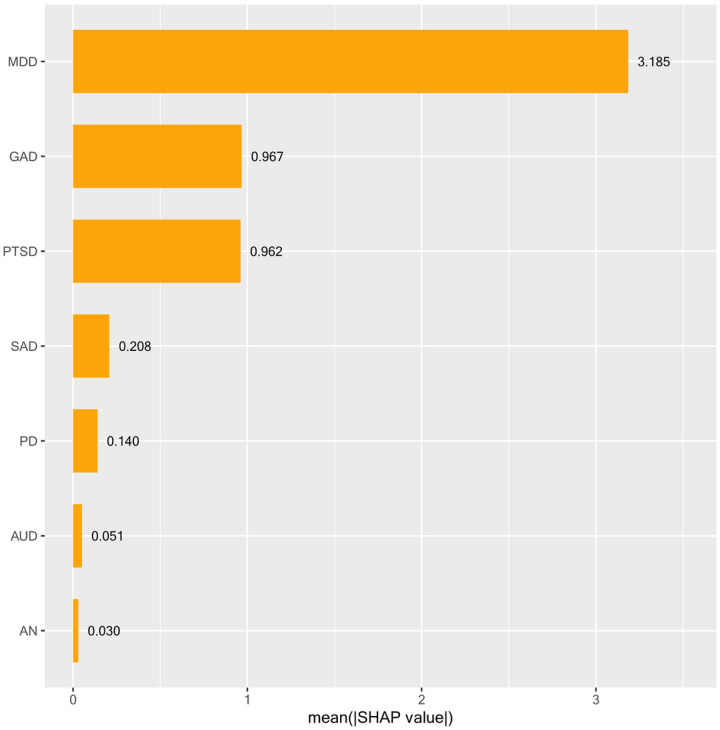
SHAP feature importance *Note*. To determine predictor importance, we used a gold standard explainable AI method termed SHapley Additive explanation (SHAP). SHAP values provide a more comprehensive understanding of each feature’s contribution to the model’s predictions.

A total of 21,899 participants were included in the training models, and 9,386 participants were included in the holdout models. The elastic net model derived from repeated 10-fold cross validation and run on the full training sample was associated with an optimal alpha parameter of .1 and a lambda parameter of .008 (via RMSE criterion). The final elastic net model from the training sample demonstrated clinical significance in predicting insomnia [R^2^ = .449 (.016), RMSE of 5.00 (.081)], with comparable variance explained in the holdout sample (i.e., completely unseen new cases) (R^2^ = .33, RMSE of 5.47). Results of the feature importance analysis are displayed in Fig. 1, in which SHAP values illustrate that MDD (SHAP = 3.185) was the most important feature associated with insomnia, followed by GAD (SHAP = 0.967) and PTSD (SHAP = 0.962).[1] Across all predictors, the presence of any psychiatric disorder was associated with higher insomnia severity, with major depressive disorder demonstrated to be the most associated disorder.

### Directed acrylic graphs

**Figure 2:**
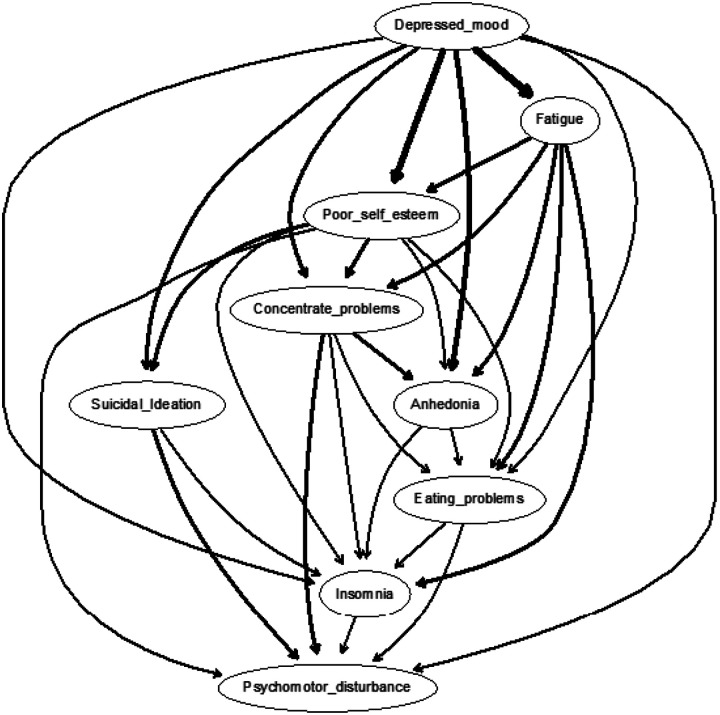
CPDAG importance *Note*. Built on train sample (N=21,899). Arrow thickness denotes a change in the Bayesian Information Criterion (BIC; a relative measure of a model’s goodness-of-fit) arising from the proportion of the averaged 10,000 bootstrapped networks wherein that arrow is removed from the network. In other words, the more an arrow contributes to the model fit, the thicker it is.

**Figure 3:**
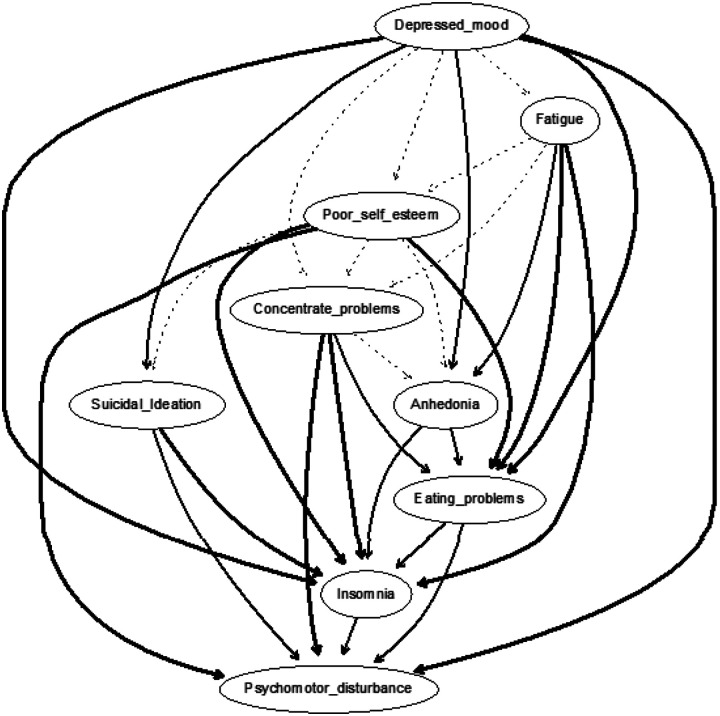
CPDAG directional probability *Note*. Built on train sample (N=21,899). Edge thickness signifies directional probabilities arising from the proportion of the averaged 10,000 bootstrapped networks wherein that arrow was pointing in that direction, or, in other words, confidence that the direction of prediction flows in the direction depicted in the graph.

The CPDAG built on training data (N = 21,899), as displayed in Fig. 2, shows a chain of symptoms dependent on the parent node of depressed mood, which directly predicted fatigue, anhedonia, poor self-esteem, concentration problems, eating problems, psychomotor disturbance, suicidal ideation, and insomnia. That is, depressed mood had no incoming edges (i.e., in-degree = 0) but had eight outgoing edges (i.e., out-degree = 8). The most important arrows connected depressed mood to fatigue (with a change in BIC of −4067.813) and depressed mood to poor self-esteem (with a change in BIC of −3294.177). Accordingly, fatigue emerged as a key step in the cascading node with one incoming arrow (i.e., in-degree = 1) and five direct descendants (out-degree = 5): anhedonia, poor self-esteem, concentration problems, eating problems, and insomnia. There were seven total paths for insomnia (depressed mood, fatigue, anhedonia, poor self-esteem, concentration problems, eating problems, and psychomotor disturbance). In other words, all depression symptoms, except for suicidality, presupposed insomnia. That is, insomnia was more likely when depressed mood, fatigue, anhedonia, poor self-esteem, concentration problems, eating problems, and psychomotor disturbance were present than vice versa. Suicidality occurred only through depressed mood, poor self-esteem, and psychomotor disturbance. This could have arisen from eating problems or concentration problems, and depressed mood and poor self-esteem. Suicidality and insomnia were the only symptoms without any descendants and, thus, were not a prerequisite for any other symptoms. Additional DAGs with arrow thickness denoting directional probability using Sachs et al.’s [80] approach was also run. As seen in Fig. 3, descendants from depressed mood to fatigue occurred only in 50.525% and depressed mood to self-esteem in 55.710% of the 10,000 networks.

### Structural Distance

**Figure 4:**
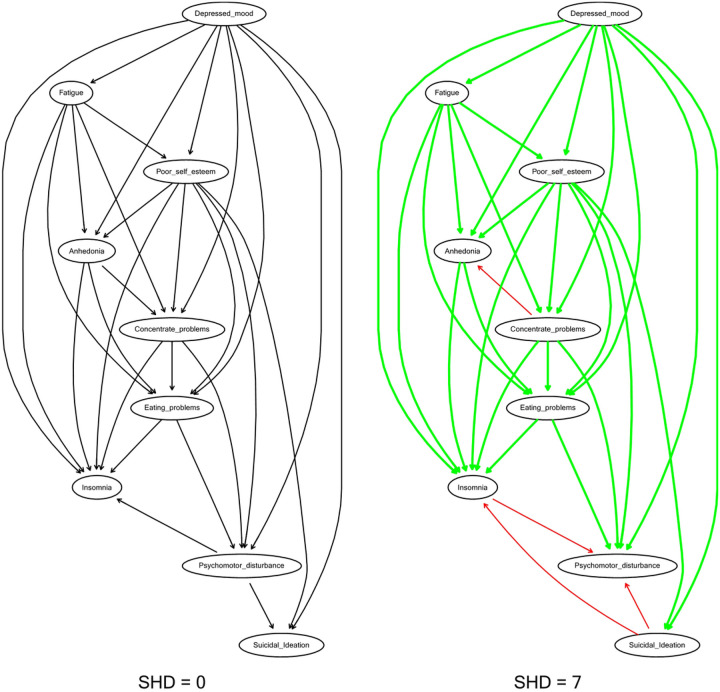
Replicability of CPDAG between train and holdout samples *Note*: train sample (N=21,899); holdout sample (N = 9,386). Green arrows = true positives; Red arrows = false positives; SHD = structural hamming distance. SHD assesses similarity between two CPDAGS and represents the number of edge insertions, deletions or flips to transform one graph to another graph. Lower values represent higher similarity.

To further facilitate the stability of our findings, we ran a second CPDAG network (as shown in Fig. 4) within our holdout data (N = 9386) using the same procedures within the training network and computed structural distances between the two networks. SHD between the training and holdout CPDAGs was low (SHD = 7), indicating an excellent fit. The parent node of depressed mood and fatigue, as a cascading node, along with its five direct descendants, anhedonia, poor self-esteem, concentration problems, eating problems, and insomnia, remained the same across networks. However, there were false positive directions in which directions switched within the holdout network as compared to the training network, or the “true network”. These arrows were concentration problems related to anhedonia and insomnia related to psychomotor disturbance. Accordingly, within the holdout network, insomnia attained one direct descendant, signifying that psychomotor disturbance was more likely when insomnia was present than vice versa. Suicidality also gained two descendants: psychomotor disturbance and insomnia. In other words, suicidality occurred only through depressed mood or poor self-esteem and directly predicted insomnia and psychomotor disturbance. Thus, nodes without any descendants switched from suicidality and insomnia to psychomotor disturbance within the holdout sample, implying that suicidality was not a prerequisite for other symptoms.

## Discussion

The present study set out to investigate the associations between insomnia and multiple psychiatric disorders within a large sample of nationally representative treatment-seeking U.S. college students. To do so, we implemented a three-step modeling approach using machine learning and Bayesian network analysis to (a) determine which psychiatric disorders were associated with insomnia, (b) tease apart symptom-level interactions of disorders most associated with insomnia, and (c) evaluate replicability for both models.

Given our interest in predicting insomnia outcomes, we used a broad range of mood, anxiety, eating, and substance use disorders to predict insomnia severity using elastic net regularization. The elastic net model accounted for 33% (R^2^ = .33) of the variance in insomnia, in part due to the inclusion of MDD, which SHAP values identified as the top factor most associated with insomnia. GAD and PTSD, respectively, were also listed as secondary and tertiary predictors contributing to the model’s performance but to a lesser degree. Findings are in parallel with Bard et al. [11], who found MDD symptoms (e.g., low energy, depressive affect via PHQ-9) to be key features across multiple domains of sleep functioning and impairment as compared to anxiety [GAD-7; 82] and insomnia symptoms [SCI-9; 83]. Our results converge with McCallum et al. [84], who found GAD, MDD, and PTSD, respectively, as the top contributors to sleep disturbance, although findings switched between the first and secondary top contributors. Discrepancies may be due in part to sample differences, as we utilized a representative sample of college students in the American population as compared to McCallum et al. [5], who noted self-selection bias within their general community Australian sample. Probable measurement error, given the usage of non-validated self-report checklists based on DSM-5 criteria as compared to the present study, which used valid and reliable diagnostic self-report measures with adequate kappa agreement with structured interviews (e.g., GAD-Q-IV, SPDQ, PDSR, PC-PTSD). But also, our analytic approaches diverge from theirs, given the present study derived feature importance via the explanatory power of a machine learning model with all disorders contained in the model as compared to p values from separate regressions for each disorder tested.

DAG analyses were conducted to offer additional insight as to how MDD symptoms may have led to insomnia. Depressed mood was found to be the most important parent symptom, directly predicting fatigue, anhedonia, poor self-esteem, concentration problems, eating problems, psychomotor disturbance, suicidal ideation, and insomnia. Stated differently, the presence of fatigue, anhedonia, poor self-esteem, concentration problems, insomnia, eating problems, and psychomotor disturbance all presupposed the presence of depressed mood more than vice versa. In a typical DAG structure, higher upstream nodes are given greater predictive priority, whereas downstream nodes carry less activation potential and are less likely to influence other symptoms in the network. These findings suggested that insomnia was seemingly dependent on other downstream symptoms in the network, indicating that the occurrence of insomnia more likely depended on the presence of MDD symptoms rather than vice versa. Notably, network estimation related to parent nodes was consistent across both training and holdout samples, further suggesting replicability. However, caution is warranted when inferring nodes with no descendants (i.e., not a prerequisite for other symptoms) as discrepancies between samples were observed. Future simulation studies are needed to determine the typical conditions when differences in network estimations arise between data subsets and their implications on validity.

Nonetheless, our findings are consistent with DSM-5 guidelines on MDD [2], suggesting that depressed mood is a hallmark feature of MDD and is one of the two symptoms required for assigning a positive diagnosis [85]. Moreover, findings of depressed mood as a parent symptom aligned with extant network reviews on MDD [50, 86–88], investigations that set out to identify the most important central symptoms of MDD [e.g., 89, 90–92], and those associated with insomnia [24, 27, 40, 47]. Insomnia is commonly found to be a robust risk factor for both first episode and recurrent depressive episodes [93]. Mechanistically speaking, Harvey [94] denoted that such associations occurred due to the presence of a bidirectional cycle. Disturbances in mood and symptoms during the day disrupt nighttime sleep, whereas sleep deprivation worsens mood regulation and symptoms the following day, creating a vicious cycle. Such cycles further persist, given that individuals with mood disorders are vulnerable to disruptions in biological rhythms and that external stressors can lead to such disruptions in biological rhythms [95]. Accordingly, college populations may be prone to such cycles, considering their increased physiological changes, heavy academic workload, and psychosocial stressors [96].

DAG analyses also implicated the presence of insomnia as probabilistically dependent on the presence of both fatigue and poor self-esteem. These findings are in line with existing centrality findings of depressed mood, fatigue, and self-esteem symptoms emerging across Western [90, 97–99] and Eastern cultures [100–102]. Furthermore, other findings implicated depressed mood directly leading to fatigue [47] or indirectly impacting insomnia through fatigue [103]. In fact, fatigue has been reported as the highest bridge symptom linking depression and insomnia symptom communities [25, 26, 38, 104].

Our findings also provide implications for treatment targets among patients with comorbidities. Results suggested that the interrelationships of depressed mood, fatigue, and self-esteem presupposed insomnia. Untreated insomnia or depression in patients with both disorders has been shown to maintain the risk of relapse due to its link with mood dysregulation [94, 105]. As such, the presence of both disorders should be assessed during population-level screening and patient management. However, and notably, depression treatment does not synonymously equate to ameliorating insomnia, e.g., sleep-related complaints are often the most common residual symptoms after antidepressant treatment [106], warranting targeted insomnia treatment. Interventions for insomnia often necessitate specific behavioral strategies (e.g., sleep hygiene), which are not constituted in pharmacotherapy and traditional CBT. Studies treating either insomnia (e.g., with CBT-I) prior to depression (e.g., with escitalopram) or vice versa have demonstrated greater improvements in insomnia and depressive symptoms compared to treatment of depression alone [for review, see 106]. However, concomitant approaches (i.e., treating both insomnia with CBT-I and depression with antidepressants at the same time) led to mixed results with inconclusive significant differences in improvements compared to treating depression alone [107–109]. As CBT-I has also been shown to be effective in treating both insomnia and depressive symptoms among those who have both, further randomized clinical trials are needed to determine if treatment combinations are better than either approach alone, for example, evaluating treatment efficacy comparing CBT-I and CBT for depression to CBT-I first vs. CBT for depression.

The current study is not without caveats and deserves careful consideration. All analyses were based on observational and exploratory data rather than experimental. Although Bayesian learning methods can enable probabilistic causal inferences, networks derived from such data cannot make strong inferences of causation from cross-sectional data. To make such inferences within the network paradigm requires additional assumptions [e.g., 53, 110, 111, 112]. Also, our CPDAG models rested on several key assumptions inherent to Bayesian networks, including the assumption of causal relations among symptoms and acyclicity, and that no important variables were excluded from the network. There were reasons to suspect that the acyclicity assumption may have been violated given the degree of potential reverse directionality. Here, arrows that were deemed most important seemed relatively thin, indicating that the direction of the arrow was pointing in both directions in a substantial percentage of bootstrapped networks. For example, depressed mood and fatigue almost certainly had a bidirectional influence on one another. Accordingly, the edge connecting depressed mood to fatigue pointed in that direction 50% of the 10,000 bootstrapped networks. The direction of the association between these two variables may thus have tipped in both directions, implying a possible ‘hidden’ cycle within an acyclic graph. The impact of violating the assumption of acyclicity is unknown but, at a minimum, implies the current DAG analyses failed to detect feedback loops. Hence, a major limitation of the present findings is it may only be treated as a simplified snapshot of probabilistic causal relations. Future studies could improve upon our approach by gathering time-series data that enable DAGs to detect feedback loops to elucidate the potential bidirectional dependencies between variables [e.g., Shin et al. 113].

The present study unravels associations related to insomnia and common comorbidities within a sample of U.S. treatment-seeking college students. Results illuminate MDD as the most important association with insomnia and the interrelationships of depressed mood, fatigue, and self-esteem that presupposed insomnia. These findings serve as a foundation for generating hypotheses rather than conclusive, causal evidence, emphasizing the need for further research into the intricate associations among psychiatric disorders in college populations. The presented modeling approach to combining supervised machine learning and Bayesian network analysis may be valuable to tease apart directionality when developing prediction models.

## Figures and Tables

**Table 1:** Sample distribution across demographic characteristics

		*n*	%
Sex (at birth)	Male	9950	31.8%
	Female	20597	65.8%
Gender	Male	9798	31.3%
	Female	19823	63.4%
	Other	1664	5.3%
Sexual orientation	Heterosexual	22759	72.7%
	Lesbian/Gay	1002	3.2%
	Bisexual	3453	11.0%
	Other	3211	10.3%
Race	White	20563	65.7%
	Black or African American	1992	6.4%
	American Indian or Alaskan Native	164	0.5%
	Asian	4236	13.5%
	Native Hawaiian or Pacific Islander	83	0.3%
	Multiracial	1790	5.7%
Ethnicity	Non-Hispanic	18673	67.5%
	Hispanic	3143	29.3%
Parental education	Bachelor’s degree or higher	21126	67.%
	Less than bachelor’s degree	9160	29.3%
Financial difficulty	Not Very Hard	13075	41.8%
	Somewhat Hard	9570	30.59%
	Hard	4913	15.70%
	Very hard	2833	9.1%
Year in school	Undergraduate 1	12807	40.9%
	Undergraduate 2	9014	28.8%
	Undergraduate 3	5007	16.0%
	Undergraduate 4	3725	11.9%
	Undergraduate 5	732	2.3%
School sector	Public	26783	85.6%
	Private	4502	14.4%

*Note. N* = 31,285.

## Data Availability

The datasets used and/or analyzed during the current study are available from the corresponding author on reasonable request.

## Abbreviations

ADHD: Attention Deficit Hyperactivity Disorder
AN: Anorexia Nervosa
AUD: Alcohol Use Disorder
BIC: Bayesian information criterion
CBT-I: Cognitive Behavioral Therapy for Insomnia
CPDAG: Completed partially directed acrylic graph
DAG: Directed acyclic graphs
GAD: Generalized Anxiety Disorder
GAD-Q-IV: Generalized Anxiety Disorder Questionnaire-IV
GGM: Graphical gaussian model (GGM)
ISI: Insomnia Severity Index
LASSO: Least Absolute Shrinkage and Selection Operator
MDD: Major Depressive Disorder
OCD: Obsessive-Compulsive Disorder
PC-PTSD-5: Primary Care PTSD Screen
PD: Panic Disorder
PDSR: Panic Disorder Self-Report
PGD: Prolonged Grief Disorder
PHQ-9: Patient Health Questionnaire-9
PTSD: Post-Traumatic Stress Disorder
R^2^: Coefficient of determination
RMSE: Root mean square error
SAD: Social Anxiety Disorder
SHAP: Shapley Additive exPlanations
SHD: Structural hamming distance
SMOGN: Synthetic Minority Over-Sampling Technique for Regression with Gaussian Noise
SPDQ: Social Phobia Diagnostic Questionnaire
SUD: Substance Use Disorder
WCS: Weight and Shape Concerns Scale
